# UHMWPE-Based Glass-Fiber Composites Fabricated by FDM. Multiscaling Aspects of Design, Manufacturing and Performance

**DOI:** 10.3390/ma14061515

**Published:** 2021-03-19

**Authors:** Sergey V. Panin, Dmitry G. Buslovich, Yuri V. Dontsov, Svetlana A. Bochkareva, Lyudmila A. Kornienko, Filippo Berto

**Affiliations:** 1 Laboratory of Mechanics of Polymer Composite Materials, Institute of Strength Physics and Materials Science SB RAS, 634055 Tomsk, Russia; buslovichdg@gmail.com (D.G.B.); doncov@mail2000.ru (Y.V.D.); svetlanab7@yandex.ru (S.A.B.); rosmc@ispms.ru (L.A.K.); 2 Department of Materials Science, Engineering School of Advanced Manufacturing Technologies, National Research Tomsk Polytechnic University, 634030 Tomsk, Russia; 3 Department of Mechanical and Industrial Engineering, Faculty of Engineering, Norwegian University of Science and Technology (NTNU), 7491 Trondheim, Norway; filippo.berto@ntnu.no

**Keywords:** ultra-high molecular weight polyethylene, microfiller, compatibilizer, strength, wear resistance, supermolecular structure

## Abstract

The aim of the paper was to improve the functional properties of composites based on ultra-high molecular weight polyethylene (UHMWPE) by loading with reinforcing fibers. It was achieved by designing the optimal composition for its subsequent use as a feedstock for 3D-printing of guides for roller and plate chains, conveyors, etc. As a result, it was experimentally determined that loading UHMWPE with 17% high density polyethylene grafted with VinylTriMethoxySilane (HDPE-g-VTMS) was able to bind 5% glass fillers of different aspect ratios, thereby determining the optimal mechanical and tribological properties of the composites. Further increasing the content of the glass fillers caused a deterioration in their tribological properties due to insufficient adhesion of the extrudable matrix due to the excessive filler loading. A multi-level approach was implemented to design the high-strength anti-friction ‘UHMWPE+17%HDPE-g-VTMS+12%PP’-based composites using computer-aided algorithms. This resulted in the determination of the main parameters that provided their predefined mechanical and tribological properties and enabled the assessment of the possible load-speed conditions for their operation in friction units. The uniform distribution of the fillers in the matrix, the pattern of the formed supermolecular structure and, as a consequence, the mechanical and tribological properties of the composites were achieved by optimizing the values of the main control parameters (the number of processing passes in the extruder and the aspect ratio of the glass fillers).

## 1. Introduction

Ultra-high molecular weight polyethylene (UHMWPE) has numerous excellent characteristics including high impact strength, abrasion, and chemical resistance as well as biocompatibility [1,2,3]. Due to the high molecular weight of (3.5–7.5)·10^6^ g/mol, UHMWPE possesses a low melt flow index (MFI) of about zero, which makes it unsuitable for processing with conventional methods applied for polymers (screw extrusion, injection molding, and others) [4,5,6].

It is known that UHMWPE can be plasticized by loading with polypropylene (PP) [7,8,9,10,11], polyethylene glycol (PEG) [12,13,14,15], high density polyethylene (HDPE) [16,17,18,19,20], etc. Liu et al. [7] studied the morphology and properties of ‘UHMWPE+PP’-based composites fabricated by the extrusion of mixtures using a single screw extruder. The microstructural analysis showed that PP was localized in both amorphous and low-crystalline zones of the UHMWPE matrix. At the same time, the UHMWPE fluidity increased significantly with enhancing the PP content. Xie et al. [12] significantly reduced the viscosity of melted UHMWPE by loading with PP and PEG. The mechanism of this effect was explained by untangling polymer chains. Lim et al. [18] investigated ‘HDPE-UHMWPE’ composites with different component ratios to determine their suitability as biomaterials. The presence of HDPE in the composites enabled a marked rise in MFI. In doing so, UHMWPE possessed improved toughness. The optimum conditions for mixing the feedstocks in the extruder were established. Kuang et al. [20] improved the UHMWPE’s manufacturability by loading with poly(amido amine) dendrimer, and its derivatives with various end groups. Filling the UHMWPE matrix with dendritic modifiers resulted in a significant decrease in the melt viscosity, which allowed for an improvement in its processability.

Fibrous polymer composites are gaining widespread attention due to their low weight, high strength, and corrosion resistance. Loading with fibrous fillers enables the preservation of the original matrix characteristics and improve other operational properties [21,22,23,24,25,26,27]. The effects of loading with fiberglass (FG) and carbon fibers (CF) on the tribological properties of the UHMWPE-based composites was studied in [26]. The FG content of 10% was the optimal level to effectively reduce the wear rate and the friction coefficient of the composites. Chukov et al. [27] investigated UHMWPE-based composites containing chopped CF in amounts of up to 12%. Annealing (in air at a temperature of 500 °C) resulted in a defect structure formation on the CF surfaces. Consequently, the activity of the latter was increased. Thus, GF and CF are common fillers for UHMWPE in order to obtain high-strength wear-resistant composites fabricated by compression sintering.

It is known that in addition to the matrix characteristics, the mechanical properties of fiber-reinforced polymer composites mainly depend on: (i) the loading degree, (ii) the physical and mechanical attributes of fibers, and (iii) the morphology, activity, and area of their surfaces, which determine the interfacial bonding between the fibers and the matrix [28,29,30]. Since FG are characterized by low interfacial adhesion with UHMWPE, the aim of research is (typically) to improve this parameter through various methods [31,32]. In particular, the issue can be solved by treatment of the FG surfaces with a silane coupling agent [33]. Composites reinforced with treated GF have shown better mechanical properties (tensile strength and impact toughness) than ones loaded with untreated fillers [34]. For instance, low density polyethylene (LDPE) chemically modified with vinyltriethoxy lane (VTES) has been used (as a coupling agent) to reinforce the UHMWPE-based composites loaded with GF [35]. As a result, they were characterized by more elastic and less ductile behavior.

However, as a rule, some key issues remain outside the scope of discussions. They include: (a) the effect of a technological sizing agent applied to improve the FG dispersibility (the presence of which does not contribute to an increase in adhesion to the polymer matrix); and (b) variations of this parameter upon manufacturing of the composites by powder methods (in this case, the nature of the filler distributions is largely limited by the arrangement of the components at the ‘mixing in suspension’ stage), etc. For this reason, alternative methods should be applied to prepare feedstocks from polymer powders and FG (for example, using a twin-screw extruder [36,37]), which enables the ability to thoroughly stir them in the molten state.

In the current study, powder mixtures were compounded in a twin-screw extruder. Then, samples were fabricated by hot compression (HC) and 3D printing (more precisely, by the fused deposition modeling (FDM)) methods using the milled granulate as a feedstock. Due to the almost zero MFI of molten UHMWPE, an additional ’17% HDPE-g-VTMS+12 % PP’ plasticizing component was loaded (hereinafter, the percentages are by weight). For the deployed twin-screw extruder, the polymer mixing parameters have already been optimized in previous studies by the authors [38]. Thus, the main difference between this paper and others devoted to the UHMWPE-based glass-fiber composites is the design of an improved one manufactured by 3D printing, which possesses high tribological and mechanical properties.

In addition, the role of compatibilizers (grafted HDPE in the studied case) should be noted, which are typically used to combine various polymers [39,40]. Loading the composites with HDPE-g-VTMS aims to provide a chemical bond between HDPE, UHMWPE, and the FG filler. According to the authors’ idea, compounding the components in the twin-screw extruder should have ensured the maximum dispersion degree for the HDPE-g-VTMS powder characterized by coarse particles.

Nevertheless, design of the high-strength ‘UHMWPE+17%HDPE-g-VTMS+12%PP’-based composites, fabricated by extrusion and 3D printing, is required to expand areas of their applications.

In [41], extrudable UHMWPE-based composites were designed, which were loaded with 30% HDPE modified with 5–10% organo-montmorillonite (OMMT). They possessed high thermal and mechanical properties for the manufacturing of pipes by screw extrusion. The effect of the HDPE-g-SMA compatibilizer and the γ-(2,3-epoxy-propoxy) propyltrimethoxysilane (KH560) silane coupling agent in an amount of 3% on the thermal properties of the UHMWPE-based pipes was investigated. It was found that loading with OMMT significantly improved the properties compared to those for pipes made of neat UHMWPE.

In these studies, the task was to improve the functional properties of the UHMWPE-based composites by loading with reinforcing fibers, but in one technological cycle (procedure), considering the features of processing UHMWPE by conventional (screw extrusion) or advanced (FDM) methods. Their manufacturability and characteristics should be at the neat UHMWPE levels. The authors deliberately chose milled and chopped FG fillers for the following reasons:

1. FG is common low-cost industrial filler, characterized by high technological effectiveness;
2. FG have a stable composition, shapes and the aspect ratio, which enables to reliably compare the effect of micron- and millimeter-scale fiber sizes;
3. Loading with commercially available HDPE-g-VTMS allows for an increase in interfacial adhesion with FG filler, which improves the physical and mechanical properties of the composites.

The goal was to design the optimal composition for its subsequent use as a feedstock for 3D printing of guides for roller and plate chains, conveyors, etc.

The remainder of this paper is structured as follows. Section 2 describes the methodological aspects of research related to the specifics of preparation and testing of samples. In Section 3, a comparison of the physical, mechanical and tribological properties is reported for the ‘UHMWPE+17%HDPE-g-VTMS+12%PP’-based composites loaded with GF of various sizes and fabricated by hot compression (HC) of powder mixtures and granules after twin-screw compounding (TSC). The final part of the third experimental section presents the results of the mechanical and tribological tests (under various load-speed conditions) of the extrudable UHMWPE-based composites fabricated by HC of the powders and granules as well as by 3D printing. In Section 4, the requirements for the composites are substantiated on the assumption of their application for guide manufacturing. These suggestions are based on the experimental results and the characteristics provided by manufacturers of the UHMWPE-based materials. In Section 5, values of the control parameters are reported, which are based on the obtained experimental data and the developed computer-aided algorithm to justify the recommendations (the number of processing passes and the aspect ratio of the GF fillers) for the manufacturing of ‘UHMWPE+17%HDPE-g-VTMS+12%PP’-based composites with the predefined mechanical and tribological properties.

## 2. Materials and Methods

### 2.1. Fabrication of the Ultra-High Molecular Weight Polyethylene (UHMWPE)-Based Composites

**Figure 1:**
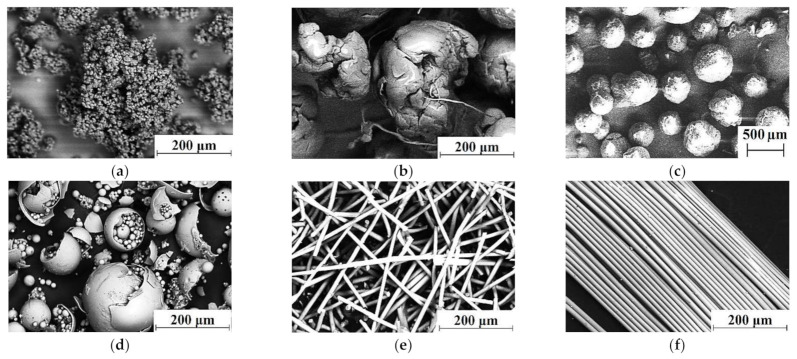
Scanning electron microscopy (SEM) micrographs of the materials: (**a**) Ultra-high molecular weight polyethylene (UHMWPE); (**b**) high density polyethylene grafted with VinylTriMethoxySilane (HDPE-g-VTMS); (**c**) polypropylene (PP); (**d**) hollow glass spheres (HGS); (**e**) milled glass fibers (MGF); (**f**) chopped glass fiber (CGF).

‘Ticona GUR-2122’ UHMWPE powder (Celanese Corporation, Irving, TX, USA) was used to fabricate samples. Its molecular weight was about 4.5 million. Particle sizes were 5–15 µm, which were weakly agglomerated into aggregates with sizes of 120–150 µm (Figure 1a). The ‘Olenten’ HDPE-g-VTMS (Figure 1b) was loaded as a compatibilizer (New Polymer Technologies LLC, Moscow, Russia). It was purchased in the form of granules 2–3 μm in size. Then, HDPE-g-VTMS was mechanically milled using a ‘Rondol’ chopper (Rondol, France) to a particle size of ~525 μm. The ‘PP 21030’ (TomskNefteChim LLC, Tomsk, Russia) particle sizes were also about 525 μm (Figure 1c). Its MFI was 3.0 g/10 min (2.1 kg). Data on GF fillers (Figure 1d–f) are presented in Table 1.

The powders and the GF fillers were mixed by dispersing the suspension in alcohol using a ‘PSB-Gals 1335-05’ ultrasonic cleaner (‘PSB-Gals’ Ultrasonic equipment center, Moscow, Russia). The processing duration was 3 min; the generator frequency was 22 kHz. After mixing, the suspension was dried in a ‘Memmert UF 55’ oven (Binder, Tutlingen, Germany) using forced ventilation at a temperature of 120 °C for 3 h.

For the purpose of homogeneous mixing of small UHMWPE particles (tens of microns in size) with large ones of the polymer fillers (hundreds of microns) for 3D-printing, they were compounded in the ‘Rondol’ twin-screw extruder. Furthermore, bulk preforms of the composites were fabricated in the following ways:

1. By HC of the multi-component powder mixtures (referred to as ‘HC-PM’ in the paper) at a pressure of 10 MPa and a temperature of 200 °C using a laboratory setup based on a ‘MS-500’ hydraulic press (NPK TekhMash LLC, Moscow, Russia). The setup was equipped with an open-loop ring furnace with a digital temperature controller (ITM LLC, Tomsk, Russia). After exposing under pressure, the preforms were cooled without unloading for 30 min. Cooling rate was 5 °C/min.
2. By HC of granules of the multi-component mixtures compounded by the Twin-Screw Extruder (referred to as ‘HC-TSE’ hereinafter). The same facilities and the parameters were applied as in the HC-PM procedure.
3. By FDM from granules of the same polymer components (referred to as ‘FDM-TSE’) using an ‘ArmPrint-2’ laboratory (craft) 3D–printer (NR TPU, Tomsk, Russia) equipped with a single-screw micro-extruder (a nozzle diameter of 0.4 mm). It was developed and deployed for printing with granules of 2–5 mm in size. A print head moved in three *XYZ* coordinates. The amount of the fed material was determined by the micro-screw rotational speed. The 3D-printer was equipped with a heated bed operated in a temperature range of 50–300 °C. The material temperature in the micro-extruder could be varied within 150–420 °C. The process was controlled by the ‘LINUX CNC’ operating system. Printing was carried out according to the model prepared in the ‘G-code’ format. Digital model files were created using ‘Repetir-Host V2.1.3’ software (Hot-World GmbH KG Knickelsdorf 4247877 Willich Germany) and ‘Slic3r’ slicer (licensed under the GNU Affero General Public License, version 3). Temperatures of the bed as well as the upper and lower regions of a filament (granules) feeder were constant at 90, 160, and 200 °C, respectively. Each deposited layer was 0.3 mm thick.

### 2.2. Examination of the Physical and Mechanical Properties

The Shore D hardness was determined using an “Instron 902” facility (Instron, Norwood, MA, USA) in accordance with ASTM D 2240. Tensile properties of ‘dog-bone’ shaped specimens were assessed using an ‘Instron 5582’ electromechanical testing machine (Instron, Norwood, MA, USA). The number of each type specimen was at least four.

### 2.3. Assessment of the Tribological Characteristics

Wear resistance was evaluated according to the ‘block-on-ring’ scheme using a ‘2070 SMT-1’ friction testing machine (Tochpribor Production Association, Ivanovo, Russia). Loads (P) on the samples were 60 and 140 N (contact pressures P_max_ of 9.7 and 32.4 MPa, respectively). Sliding speeds (V) were 0.3 and 0.5 m/s. The P·V combinations of 60 N·0.3 m/s and 140 N·0.5 m/s were designated hereinafter as the ‘mild’ and ‘severe’ tribological conditions, respectively. A disk-shaped counterpart was made of the outer ring of a commercial bear. Its diameter was 35 mm and width was 11 mm. The counterpart surface roughness was 0.20–0.25 µm. The counterpart temperature was measured using a ‘CEM DT-820’ non-contact InfraRed (IR) thermometer (Shenzhen Everbest Machinery Industry Co. Ltd., Shenzhen, China). Wear rates were determined by measuring the width and depth of the wear track according to stylus profilometry, followed by multiplication by its length. Wear rates were calculated considering the load and sliding distance values:

(1) $$\mathrm{Wear}\;\mathrm{rate}\;=\;\frac{\mathrm{volume}\;\mathrm{loss}\;\left({\mathrm{mm}}^{3}\right)}{\mathrm{load}\;\left(N\right)\times \mathrm{sliding}\;\mathrm{distance}\;\left(m\right)}$$

The wear track profiles were assessed using the data on at least ten tracks. Then, wear rates were estimated on the basis of the experimental results over at least four samples of each type. Mathematical statistics methods were applied for data processing.

### 2.4. Structural Studies

The topography of the wear track surfaces was investigated using a ‘Neophot 2’ optical microscope (Carl Zeiss, Oberkochen, Germany) equipped with a ‘Canon EOS 550D’ digital camera (Canon Inc., Tokyo, Japan), and an ‘Alpha-Step IQ’ stylus profiler (KLA-Tencor, Milpitas, CA, USA).

Initially, the notched polymer samples were cooled in liquid nitrogen at −197 °C for one hour, and then mechanically fractured. The cleaved surfaces were used to study the filler distribution as well as the supermolecular structure. In vacuum, copper films about 10 nm thick were deposited on the fracture surfaces using a ‘JEOL JEE-420’ vacuum evaporator (JEOL USA, Inc., Peabody, MA, USA). The requirements for the thickness of the conductive film were dictated by the need to preserve the morphology of the original fracture surfaces. A ‘LEO EVO 50’ scanning electron microscope (Carl Zeiss, Oberkochen, Germany) was employed at an accelerating voltage of 20 kV.

## 3. Results and Discussion

### 3.1. The Glass Powder (Hollow Glass Spheres)

In these studies, the glass powder was loaded not with the aim of improving the physical, mechanical, and tribological properties of the ‘UHMWPE+17%HDPE-g-VTMS+12%PP’-based composites, but as a ‘zero’ point, namely as a filler with the aspect ratio of 1. This was due to the facts that (i) the powder particles were too different in sizes; (ii) they were hollow and, therefore, damaged upon the mixture extrusion; and (iii) the distribution of the HGS fragments as well as size dispersion were uneven.

Table 2 presents the mechanical properties of the ‘UHMWPE+17%HDPE-g-VTMS+12%PP’-based composites fabricated by the HC-PM and HC-TSE methods at HGS loading from 2.5 to 10.0%.

Neat UHMWPE was characterized by high elongation at break values (about 485% depending on the fabrication method). During the tests, the samples were lengthened, which was accompanied by strengthening due to the predominant orientation of polymer chains. As a result, the tensile strength values were almost twice the yield strength levels. Loading UHMWPE with GF improved yield strength, especially under conditions of increased interphase adhesion. However, GF fractures during the tests caused the entire composite failures. Thus, the decrease in tensile strength was due to the small contribution of the polymer matrix to hardening. The uniform filler distribution in the matrix upon twin-screw compounding did improve tensile strength.

**Figure 2:**
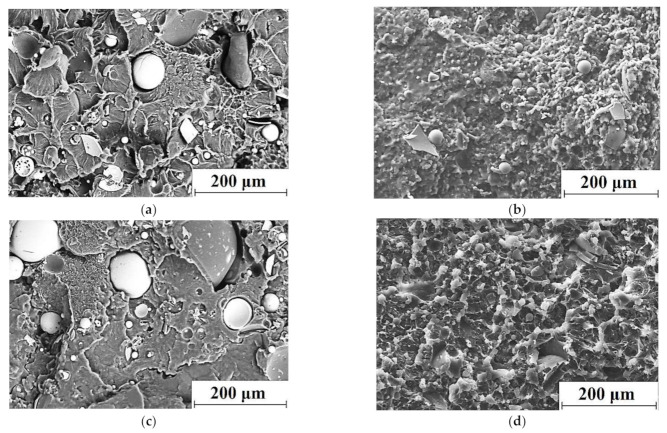
The scanning electron microscopy (SEM) micrographs of the supermolecular structure of the ‘UHMWPE+17%HDPE-g-VTMS+12%PP+5%HGS’ (**a**,**b**) and ‘UHMWPE+17%HDPE-g-VTMS+12%PP+10%HGS’ (**c**,**d**); HC-PM (**a**,**c**), hot compression by the Twin-Screw Extruder (HC-TSE) (**b**,**d**).

It follows from Table 2 that the mechanical properties (Young’s modulus and ultimate tensile strength) of the HC-PM composites were ~1.3–1.5 times lower than those for the HC-TSE ones. Figure 2 shows the SEM micrographs of the supermolecular structure of the ‘UHMWPE+17%HDPE-g-VTMS+12%PP’-based composites fabricated by the HC-PM and HC-TSE methods. The supermolecular structures of the composites fabricated by the HC-TSE procedure were fundamentally different from the HC-PM ones. The GF filler was more evenly distributed in the HC-TSE matrices. Twin-screw compounding of the multi-component polymer mixtures with different sizes of the initial components formed the homogeneous supermolecular structures of the composites (Figure 2b,d), which provided their higher mechanical properties (Table 2).

**Figure 3:**
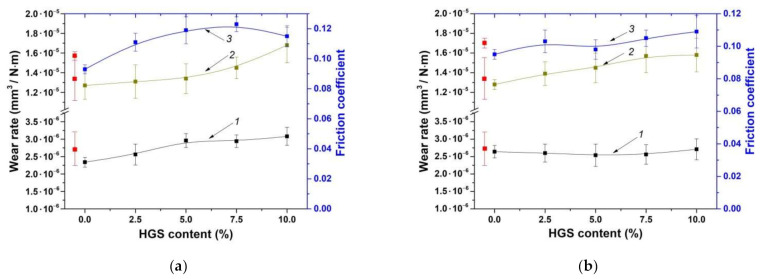
Wear rates according to the ‘pin-on-disk’ (curve 1, 5 N·0.3 m/s) and ‘block-on-ring’ (curve 2, 60 N·0.3 m/s) schemes as well as the average friction coefficients (curve 3) versus filler weight fraction: HC-PM (**a**), HC-TSE (**b**).

It is known that the mechanical and tribological properties were not always unambiguously correlated with each other. For this reason, the tribological characteristics of the HC-PM composites were also evaluated (despite their physical and mechanical properties were markedly lower). Figure 3 shows the wear rates and the friction coefficients for the ‘UHMWPE+17% HDPE-g-VTMS+12% PP’-based composites, fabricated by the HC-PM and HC-TSE methods, under the dry friction conditions according to the ‘pin-on-disk’ and ‘block-on-ring’ schemes. The friction coefficients were weakly dependent on both filler contents and fabrication methods. Thus, the results of the tribological tests should be interpreted not only on the basis of their structures and the mechanical properties.

As follows from Figure 3 (curves 1 and 3), wear rates were slightly enhanced with an increase in the HGS content. With regard to wear rates and the friction coefficient of the HC-TSE composites, almost unchanged linear trends could be observed with an increase in the filler contents.

Figure 3 (curve 2) illustrates the wear rates of neat UHMWPE and the ‘UHMWPE+17%HDPE-g-VTMS+12%PP’-based composites under the ‘mild’ tribological conditions according to the ‘block-on-ring’ scheme. Wear rates were slightly raised with the increase in the HGS content. Nevertheless, the dynamics for both HC-PM and HC-TSE composites were comparable in this case. The authors suggested that gradual wear rate enhancing for the composites substantially loaded with HGS (7.5–10%) was associated with the abrasive wear of the counterpart and its subsequent destructive effect on the surfaces of the tribological contact parts.

Thus, very heterogeneous composites had been formed by the HC-PM procedure, which could not possess high physical, mechanical, and tribological properties. However, it was possible to evenly disperse the fillers in the UHMWPE matrix by the HC-TSE method, thereby increasing the strength properties and maintaining the tribological characteristics at the neat UHMWPE level.

### 3.2. Milled Glass Fibers (200 µm in Length)

Table 3 presents the mechanical properties of neat UHMWPE and the ‘UHMWPE+17% HDPE-g-VTMS+12% PP’-based composites loaded with 2.5 to 10.0% MGF (the aspect ratio of 20) and fabricated by the HC-PM and HC-TSE methods.

It follows from Table 3 that loading with MGF into the ‘UHMWPE+17% HDPE-g-VTMS+12%PP’ matrix increased the Young’s modulus by 1.3–1.6 times compared to that for neat UHMWPE. Yield strength varied slightly with an increase in the MGF contents for the composites fabricated by both HC-PM and HC-TSE methods. Ultimate tensile strength of the HC-TSE composite loaded with 5% MGF was 1.3 times higher than that of the HC-PM one.

**Figure 4:**
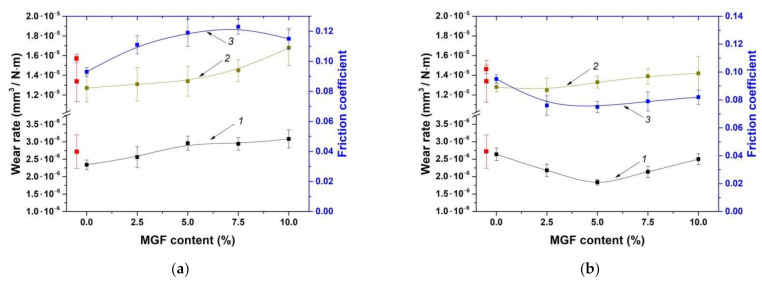
Wear rates according to the ‘pin-on-disk’ (curve 1, 5 N·0.3 m/s) and ‘block-on-ring’ (curve 2, 60 N·0.3 m/s) schemes as well as the average friction coefficients (curve 3) versus filler weight fraction: HC-PM (**a**), HC-TSE (**b**).

Figure 4 shows the friction coefficients and wear rates of the composites loaded with various MGF contents and fabricated by the HC-PM and HC-TSE methods.

It can be concluded that the wear rates of the composites fabricated by both procedures decreased with an increase in the MGF content up to 5%, and then enhanced for the HC-TSE samples. However, they did not change in the case of the HC-PM ones. Dependences in the friction coefficients from the MGF contents were similar. According to the authors, the increase in wear rate of the composites loaded with MGF was due to the imposition of the cutting action of the rough counterpart surface and the microabrasive effect of the hard FG debris.

Figure 4 (curve 2) shows the wear rates of the composites according to the ‘block-on-ring’ scheme under the ‘mild’ tribological conditions. The values enhanced with an increase in the MGF contents for the HC-PM composites. For the HC-TSE samples, wear rates were less than that for neat UHMWPE. In addition, they were at approximately the same level regardless of the MGF contents.

### 3.3. Chopped Glass Fibers (3 mm in Length)

Table 4 presents the mechanical properties of the ‘UHMWPE+17%HDPE-g-VTMS+12%PP’-based composites fabricated by the HC-PM and HC-TSE methods at CGF loading of 2.5 to 10.0% (the aspect ratio of 300).

It could be concluded that density, hardness, and Young’s modulus increased in contrast with those for the ‘UHMWPE+17%HDPE-g-VTMS+12%PP’ composite with enhancing the CGF content. In addition, elongation at break decreased by an order of magnitude for the HC-PM composites. At the same time, it only halved for the HC-TSE ones. Additionally, the Young’s modulus increased by 1.7 times over that for the non-enforced matrix in this case.

**Figure 5:**
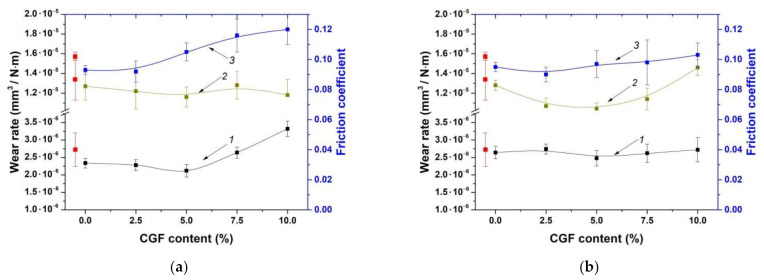
Wear rates according to the ‘pin-on-disk’ (curve 1, 5 N·0.3 m/s) and ‘block-on-ring’ (curve 2, 60 N·0.3 m/s) schemes as well as the average friction coefficients (curve 3) versus filler weight fraction: HC-PM (**a**), HC-TSE (**b**).

For both HC-PM and HC-TSE methods, the lowest wear rate was observed for the samples loaded with 5% CGF (Figure 5). With enhancing the CGF contents, wear rates increased. The dynamics of the friction coefficients was similar. These results enabled us to conclude that the CGF loading degree over 5% was accompanied by significant counterpart wear. This, in turn, caused microabrasive wear of the composites. For this reason, wear rate increased by 30% for the composites loaded with 10% CGF compared to that for the samples containing 5% CGF.

Figure 5 (curve 2) shows the wear rates of the composites under the ‘mild’ tribological conditions according to the ‘block-on-ring’ scheme. In this case, the data testified that the HC-TSE composite loaded with 5% CGF possessed the best wear resistance.

**Figure 6:**
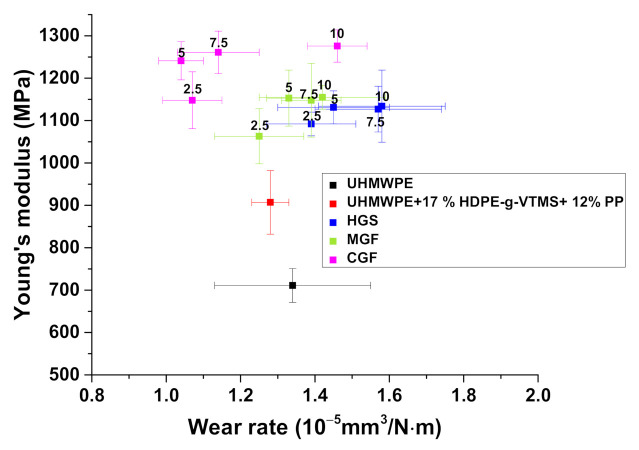
Young’s modulus vs. wear rate (the ‘block-on-ring’ scheme; 60 N·0.3 m/s); HC-TSE.

Figure 6 presents dependencies of the Young’s modulus and wear rates from the HGS, MGF, and CGF contents in the TSE composites. It follows from these data that the Young’s modulus was enhanced and the wear rates also slightly rose with an increase in the HGS content. The inexpediency of loading with more than 5% HGS could be concluded. Compared to neat UHMWPE, this improved the mechanical properties (Young’s modulus by 1.6 times and yield strength by 1.2 times) while maintaining wear resistance and ensuring the sufficient extrudability level (including for additive manufacturing). With an increase in the MGF content up to 5%, the Young’s modulus increased up to 1200 MPa with slightly enhanced wear rates. Further increase of the HGS content was not accompanied by an increase in the Young’s modulus.

With a rise in the CGF content from 5 up to 10%, the Young’s modulus was enhanced by 1.7 and 1.4 times compared to neat UHMWPE and the ‘UHMWPE+17%HDPE-g-VTMS+12%PP’ matrix, respectively. However, the increase in the CGF content up to 10% was also accompanied with a rise in wear rates by 1.5 times (from 1.04 up to 1.46·10^−5^ mm^3^/N·m) and a slight increase in Young’s modulus. Based on the obtained data, the composites loaded with 5% CGF were recommended for fabrication by the FDM-TSE method.

The results of the tribological tests of the ‘UHMWPE+17%HDPE-g-VTMS+12%PP’-based composites showed that the optimal GF content was 5% for all studied fillers (HGS, MGF, and CGF). This composition provided the improved strength properties of the multi-component extruded matrix, high wear resistance of the composites, and the necessary MFI level to be processed by screw extrusion. Then, it was used as a feedstock for 3D-printing of the composites.

### 3.4. Comparative Analysis of the Composites Fabricated by the Hot Compression (HC) and Fused Deposition Modeling (FDM) Methods

A comparative analysis was carried out to investigate the structure and characteristics of the ‘UHMWPE+17%HDPE-g-VTMS+12%PP’-based composites loaded with 5% HGS, MGF, and CGF. Table 5 presents the mechanical properties of the composites fabricated by the HC-PM, HC-TSE, and FDM-TSE methods.

**Figure 7:**
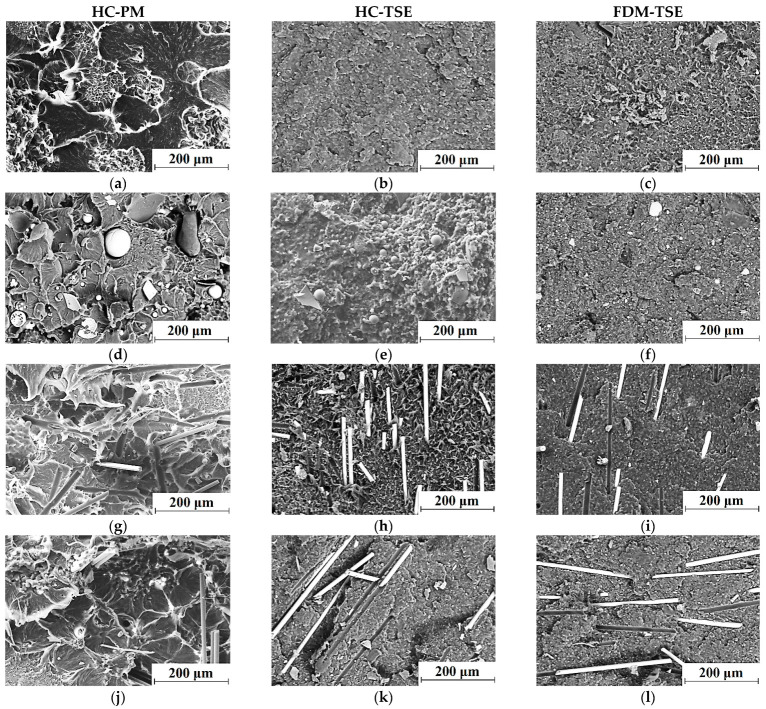
The SEM-micrographs of supermolecular structure of the ‘UHMWPE+17%HDPE-g-VTMS+12%PP’ sample (**a**–**c**) and the composites (**d**–**l**) fabricated by the HC-PM, HC-TSE, and FDM-TSE methods: ‘UHMWPE+17%HDPE-g-VTMS+12 %PP+5%HGS’ (**d**–**f**); ‘UHMWPE+17%HDPE-g-VTMS+12%PP+5%MGF’ (**g**–**i**); ‘UHMWPE+17%HDPE-g-VTMS+12%PP+5%CGF’ (**j**–**l**).

The analysis of the summarized data (see Table 5) enabled us to conclude that the mechanical properties (Young’s modulus and yield stress) of the FDM-TSE composites were 1.2–1.3 times higher than those for the non-enforced composite fabricated by the same method, and 10–15% higher than those for the HC-TSE ones. This fact could be explained by the supermolecular structures of the samples (Figure 7). The supermolecular structures of the ‘UHMWPE+17%HDPE-g-VTMS+12%PP’ matrix and the HC-PM composites included spherulites, the sizes of which depended on the aspect ratio of the GF fillers. Loading the matrix with HGS resulted in a decrease in the size of spherulites from 150 down to 50 µm (Figure 7a,d,g,j).

The supermolecular structures of the HC-TSE composites (Figure 7b,e,h,k) were fundamentally different from those of the HC-PM samples. It should be noted that both UHMWPE and HDPE-g-VTMS particles were large (hundreds of microns) for technological reasons. This fact did not facilitate their uniform distribution in the matrix. As a result of compounding in the twin-screw extruder, which implemented the development of shear strain processes in the liquid state and the subsequent solidification of the polymers with different melting points, the sizes of PP inclusions did not exceed a couple of tens of microns. Respectively, all components were uniformly distributed in the polymer composites. The glass fibers were evenly distributed in the polymer matrix. Finally, the FDM-TSE samples had even finer and more homogeneous supermolecular structures (Figure 7c,f,i,l), which corresponded to their higher strength properties in comparison with those for the HC-TSE ones.

Despite the fact that the density of the composites had decreased during the FDM layer-by-layer extrusion, the mechanical properties of the FDM-TSE composites were higher than those for the HC-TSE ones. Thus, twin-screw compounding of the multi-component polymer mixtures with different sizes of the initial components made it possible to form the homogeneous supermolecular structures of the composites and, as a consequence, to provide their higher mechanical properties. The additional single-screw extrusion of granules in the FDM procedure improved (made them finer) the elements of the supermolecular structure, further enhancing the strength characteristics of the extruded matrix and the composites. In Figure 7h,i,k,l, SEM micrographs indicate the presence of adhesion between the matrix and fibers in the composites.

**Figure 8:**
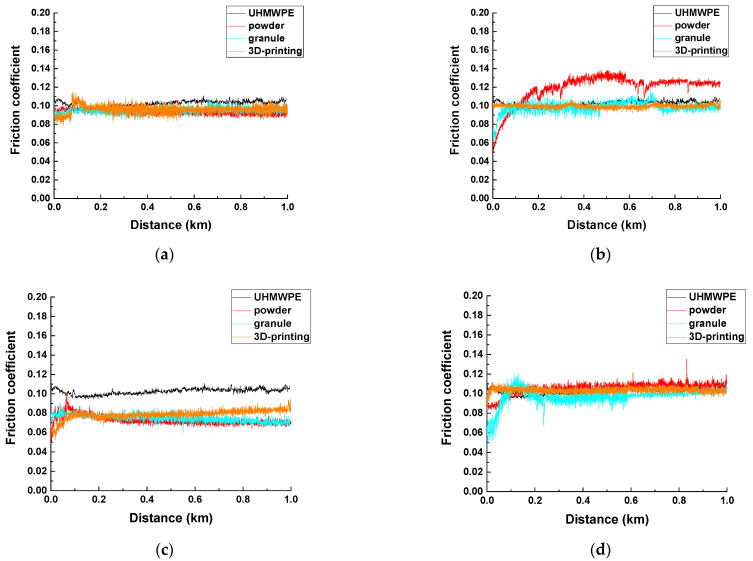
The friction coefficient vs the sliding distance: UHMWPE+17%HDPE-g-VTMS+12%PP (**a**), UHMWPE+17%HDPE-g-VTMS+12%PP+5%HGS (**b**), UHMWPE+17%HDPE-g-VTMS+12%PP+5%MGF (**c**), UHMWPE+17%HDPE-g-VTMS+12%PP+5%CGF (**d**). The ‘pin-on-disk’ scheme.

Table 6 presents the tribological characteristics of the ‘UHMWPE+17%HDPE-g-VTMS+12%PP’-based composites fabricated by all three methods under the dry sliding friction conditions according to the ‘pin-on-disk’ scheme. Their friction coefficients were significantly lower than that for neat UHMWPE, while they were weakly dependent on the sample fabrication methods. The lowest values of ~0.073–0.079 were found for the composites loaded with 5% MGF. More clearly these dependences were drawn in Figure 8. For the ‘pin-on-disk’ scheme, they were consistent with wear rates, the minimum values of which were characteristic of the composites loaded with 5% MGF (Table 6). Wear rate of the ones loaded with 5% CGF was at the level of the ‘UHMWPE+17%HDPE-g-VTMS+12% PP’ non-enforced composite.

**Figure 9:**
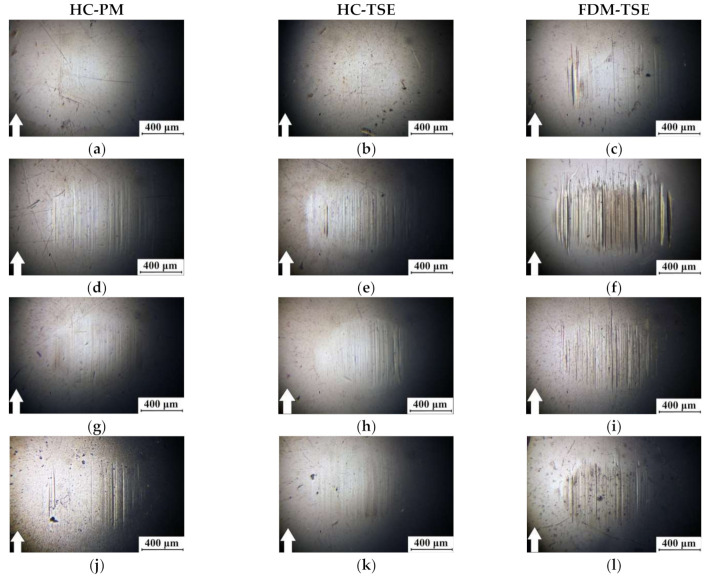
The wear track surfaces on the steel counterpart after the tribological tests on the ‘UHMWPE+17%HDPE-g-VTMS+12%PP’ sample (**a**–**c**) and the composites (**d**–**l**) fabricated by the HC-PM, HC-TSE, and FDM-TSE methods: ‘UHMWPE+17%HDPE-g-VTMS+12%PP+5%HGS’ (**d**–**f**); ‘UHMWPE+17%HDPE-g-VTMS+12%PP+5%MGF’ (**g**–**i**); ‘UHMWPE+17%HDPE-g-VTMS+12%PP+5%CGF’ (**j**–**l**). The ‘pin-on-disk’ scheme.

Figure 9 shows the optical images of the wear track surfaces of the steel counterpart after the tribological tests. Glass spheres (15–200 µm in size) and 3 mm chopped glass fibers, protruded above the polymer matrix, exerted a cutting effect on the counterpart surface to a greater extent in the FDM-TSE cases (Figure 9c,f,i).

Furthermore, the tribological characteristics, the structure, and the topography of the wear track surfaces on both the counterpart and the composites were investigated under the ‘mild’ and ‘severe’ conditions according to the ’block-on-ring’ scheme in order to determine the possible load-speed parameters for their operation in friction units (Table 7).

It follows from Table 8 that the composite loaded with 5% MGF and fabricated by the FDM-TSE method was the most efficient. Under the ‘mild’ tribological conditions, its wear rate was 0.96·10^−5^ mm^3^/N·m. Under the ‘severe’ tribological conditions, the highest wear resistance was shown by the sample loaded with 5% CGF and manufactured by the same procedure (wear rate of 1.91·10^−5^ mm^3^/N·m). Thus, the fabrication methods (the number of processing passes in the twin screw extruder) and the filler sizes (the aspect ratio) determined the predominant load–speed operation parameters of the ‘UHMWPE+17%HDPE-g-VTMS+12%PP’-based composites.

**Figure 10:**
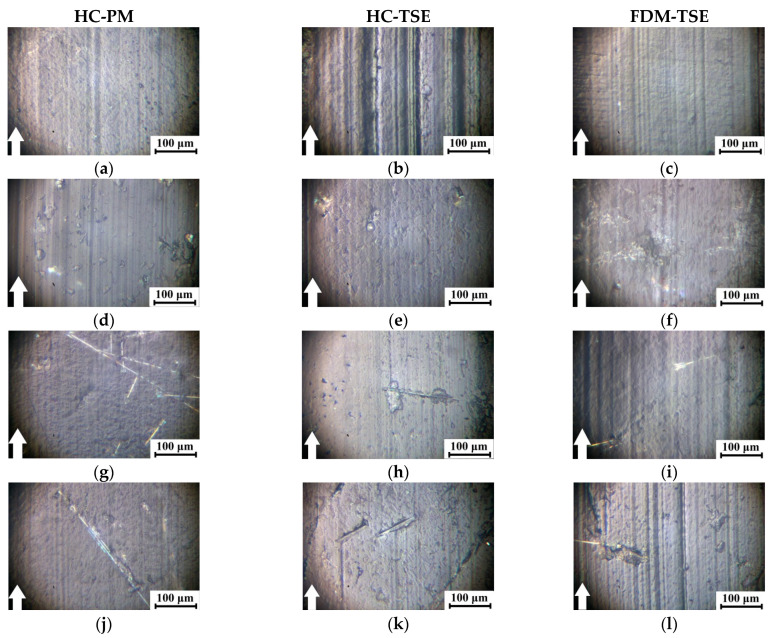
The wear track surfaces on the ‘UHMWPE+17%HDPE-g-VTMS+12%PP’ sample (**a**–**c**) and the composites (**d**–**l**) fabricated by the HC-PM, HC-TSE, and FDM-TSE methods: ‘UHMWPE+17%HDPE-g-VTMS+12%PP+5%HGS’ (**d**–**f**); ‘UHMWPE+17%HDPE-g-VTMS+12%PP+5%MGF’ (**g**–**i**); ‘UHMWPE+17%HDPE-g-VTMS+12%PP+5%CGF’ (**j**–**l**). 60 N·0.3 m/s.

**Figure 11:**
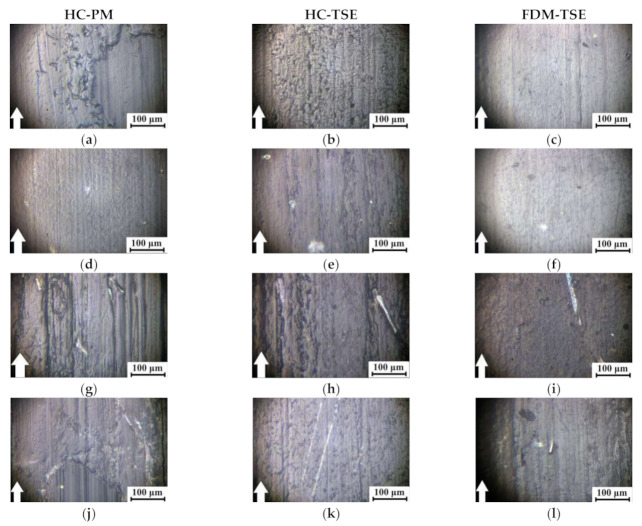
The wear track surfaces on the ‘UHMWPE+17%HDPE-g-VTMS+12%PP’ sample (**a**–**c**) and the composites (**d**–**l**) fabricated by the HC-PM, HC-TSE, and FDM-TSE methods: ‘UHMWPE+17%HDPE-g-VTMS+12%PP+5%HGS’ (**d**–**f**); ‘UHMWPE+17%HDPE-g-VTMS+12%PP+5%MGF’ (**g**–**i**); ‘UHMWPE+17%HDPE-g-VTMS+12%PP+5%CGF’ (**j**–**l**). 140 N·0.5 m/s.

Figure 10 and Figure 11 show the micrographs of the wear track surfaces on the ‘UHMWPE+17%HDPE-g-VTMS+12%PP’-based composites fabricated by the HC-PM, HC-TSE, and FDM-TSE methods. Under the ‘mild’ tribological conditions, the smoothest surface of the matrix samples was typical for the FDM-TSE case (Figure 10c). On the wear track surface of the most wear-resistant FDM-TSE composite (Figure 10i), the grooves were formed, highly likely, due to its low density (e.g., compared to the HC-TSE one, Figure 10h). In general, the topography of the wear track surfaces of all composites could be considered similar, with the exception of the FDM-TSE sample loaded with CGF. In this case, some grooves were observed on the wear track surface. According to the authors, this was due to fiber fracture and their subsequent plowing action (Figure 10l).

Under the ‘severe’ tribological conditions, wear rates of the composites varied less (the values were in the range of (1.91–2.70) 10^−5^ mm^3^/N·m). For this reason, the topography of the wear track surfaces also differed to a lesser extent. In this case, the smoothest ones were characteristic of the samples fabricated by the FDM-TSE method (Figure 11c,f,i,l).

Comparison of the topography of the wear track surfaces on the ‘UHMWPE+17%HDPE-g-VTMS+12%PP’-based composites fabricated by the HC-PM, HC-TSE, and FDM-TSE methods showed that the relief features correlated well with the tribological characteristics under both ‘mild’ and ‘severe’ tribological conditions. The observed warts on the wear track surfaces of the HC-PM samples were due to the heterogeneity of their supermolecular structure.

The wear track surfaces were smoother on the HC-TSE samples, while there was little debris adhered on the counterpart (Figure 9 and Figure 10). The FDM-TSE samples were much more flattish; however, there was quite a lot of debris on the deep-plowed counterpart surface after friction on the composite loaded with 5% CGF (Figure 9).

Thus, the dimensions of glass filler (the aspect ratio) and the fabrication methods of the UHMWPE-based composites (the amount of processing passes of the initial components in the extruder) determined the distribution of the fillers in the matrix and the supermolecular structure. As a consequence, their mechanical and tribological properties also varied. These two control parameters were used for the computer-aided design of composites with specified properties using an algorithm developed by the authors [22] to determine the acceptable load-speed ranges of their operation in friction units.

## 4. Justification of Requirements for Reinforced Ultra-High-Molecular-Weight Polyethylene (UHMWPE)-Based Composites

Using the best obtained composite, the authors proposed fabricating guides for sliding friction units. Below is a brief overview of the quantitative parameters that various manufacturers have designated as basic data for these purposes. Most companies offer a wide variety of the guide designs: for roller and plate chains, bottle guides, V-belts, conveyors, etc.

In a catalog of Misumi Group Inc. (Tokyo, Japan), the following products were offered: UHMW guide rails, UHMW guide rail shields, engineering plastic ultra-high molecular weight polyethylene irregular shaped rail, UHMW tape, wear strip (flat rail), etc. The properties of the materials for their manufacturing are presented in Table 9.

Guangzhou Engineering Plastics (Guangdong, China (Mainland)) have also developed products for similar purposes, namely: Engineering Plastics UHMWPE plastic bend guide, UHMWPE guide rail UHMWPE linear guide, UHMWPE sliding conveyor chain guide, and extruding guide plastic UHMWPE linear straight guide rail. The properties of the materials for their manufacturing are presented in Table 10.

In the catalog of Polymer Industries (Henagar, AL, USA), the Polyslick UHMW products were offered in addition to the guides: bus curb, fender pads, flexi fend, pile guards, rocket plate, UHMW rod, UHMW sheet, etc. The properties of the materials for their manufacturing are presented in Table 11.

Tangyin Dingyuan Engineering Plastics Co. Ltd. (Anyang, Henan, China) manufactured the following products: UHMW sheet, belt conveyor roller, wear resistant light and strong UHMWPE scraper, customized UHMW parts including UHMW scraper, UHMW bearing, UHMW gears, UHMW washers, and plastic chain guide rails. The properties of the materials for their manufacturing are presented in Table 12.

The Polystone^®^ M natural (PE-UHMW/PE 1000) material was produced by Röchling Engineering Plastics (Mannheim, Baden-Württemberg, Germany), which was also used as the base for the guides (Table 13).

The TIVAR^®^ 1000 (neat UHMWPE) and TIVAR^®^ 88 (glass-filled) materials, analogous to Polystone^®^ M UHMWPE were manufactured by Quadrant Engineering Plastics Products, a part of the Mitsubishi Chemical Advanced Materials group (Lenzburg, Switzerland). Their properties are presented in Table 14 and Table 15, respectively.

Murtfeldt Kunststoffe GmbH KG (Dortmund, Germany), one of the world’s leading manufacturers of slide profiles, fabricated chain guides and belts, tensioning devices, and various mechanical sliding components made of wear-resistant polymers. The properties of its Werkstoff’S′^®^ (РЕ 1000’S′) material are presented in Table 16.

Based on the above data, the authors applied the following physical, mechanical, and tribological properties of the materials as threshold values:

Bulleted lists look like this (reference data for neat UHMWPE were taken from the authors’ data):

- Tensile Young’s modulus of 1200 MPa. This level exceeded by 400–500 MPa those for all studied neat UHMWPE specimens;
- Yield strength of 25 MPa. In this case, the authors used the threshold value above that for neat UHMWPE;
- Ultimate tensile strength of 24 MPa. This level was taken slightly lower than that for neat UHMWPE, since polymer reinforcement was accompanied by the significant decrease in elongation at break, and, accordingly, in ultimate tensile strength. However, this value was high enough even for neat UHMWPE;
- Elongation at break of 200%. This threshold was chosen based on the above data (Table 1, Table 2, Table 3, Table 4, Table 5, Table 6, Table 7 and Table 8), where the minimum values ranged from 200 up to 300%. In this case, the authors proceeded from the assumption that an increase in the strength properties due to the GF reinforcement should not be accompanied by a decrease in elongation at break by more than two times in regard to that for neat UHMWPE (~485%);
- The friction coefficient of 0.102. According to the published data, the friction coefficients were weakly correlated with wear resistance. In this case, the value was chosen at the neat UHMWPE level of about 0.1 (Table 1, Table 2, Table 3, Table 4, Table 5, Table 6, Table 7 and Table 8);
- Wear rate of 1.1·10^−5^ mm^3^/N·m under the ‘mild’ tribological conditions. The threshold value was predefined to provide 20% higher wear resistance relative to that for neat UHMWPE;
- Wear rate of 2.2·10^−5^·mm^3^/N·m under the ‘severe’ tribological conditions, at which the multiple higher wear rates were observed in comparison with the previous case and should be considered as short-acting ones.

## 5. The Composite Design Algorithm for the Guides

Considering the results described in Section 3, the optimal values of the control parameters were further determined, namely (i) the number of processing passes in the extruder and (ii) the aspect ratio of the glass filler. The aim was to provide the required mechanical and tribological properties of the composites, substantiated in Section 4. For this purpose, an approach to their rational design was implemented [62,63], based on the use of a limited amount of the experimental data (Table 5, Table 6 and Table 7), which were supplemented by applying an interpolation procedure in order to obtain continuous dependences. Then, these dependencies on the composite fabrication parameters (in this case, (i) the number of processing passes in the extruder and (ii) the aspect ratio of the glass filler) were drawn in the form of isolines, on which an area with the specified restrictions was assessed. The resulting graphs of all characteristics were superimposed on each other, which limited the area met the specified requirements. The visualization of the calculated data simplified the assessment of the influence of the control parameters on the properties of the composites and made it possible to determine their specific values required for manufacturing the materials with the predefined properties.

In the studied case, the experimentally obtained properties of the composites (Table 5, Table 6 and Table 7) were determined by the control parameters: the number of processing passes in the extruder (0 for HC-PM; 1 for HC-TSE; and 2 for FDM-TSE) as well as the aspect ratio of the glass filler (1 for HGS; 20 for MGF; and 300 for CGF). The values of other parameters were constant (the loading degree of 5%, fiberglass diameters, etc.). Additional values were assessed by the interpolation [64] for intermediate points between the above-mentioned aspect ratios of the glass fillers. Distances between the nodes determining such aspect ratios were incommensurable. This resulted in large interpolation errors, even despite the use of a polynomial for unequally spaced nodes [64]. In order to change the distances between the nodes and reduce the interpolation errors, the values of the interpolation nodes were logarithmized using the natural logarithm (ln(1) = 0; ln(20) = 3, ln(300) = 5.7). The obtained data were used as the node points.

**Figure 12:**
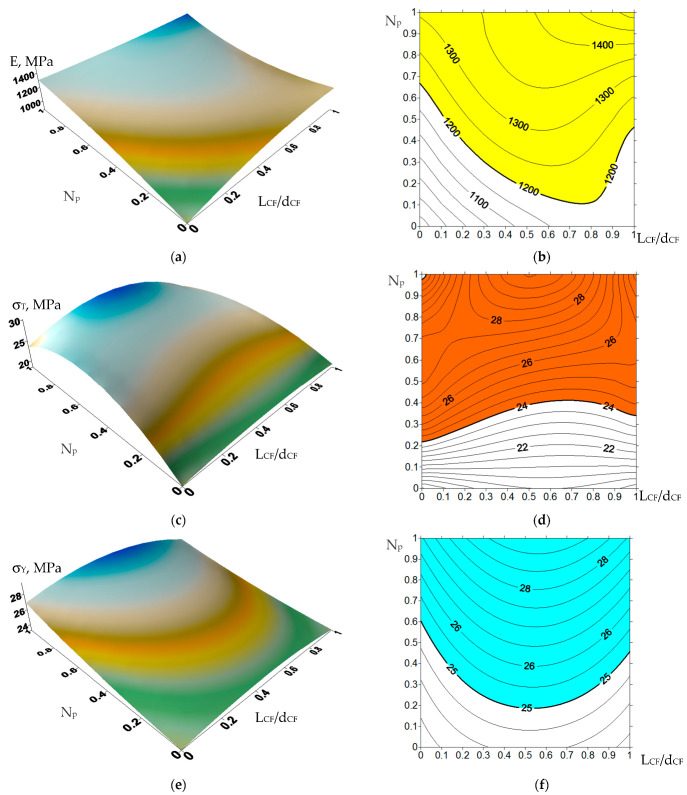

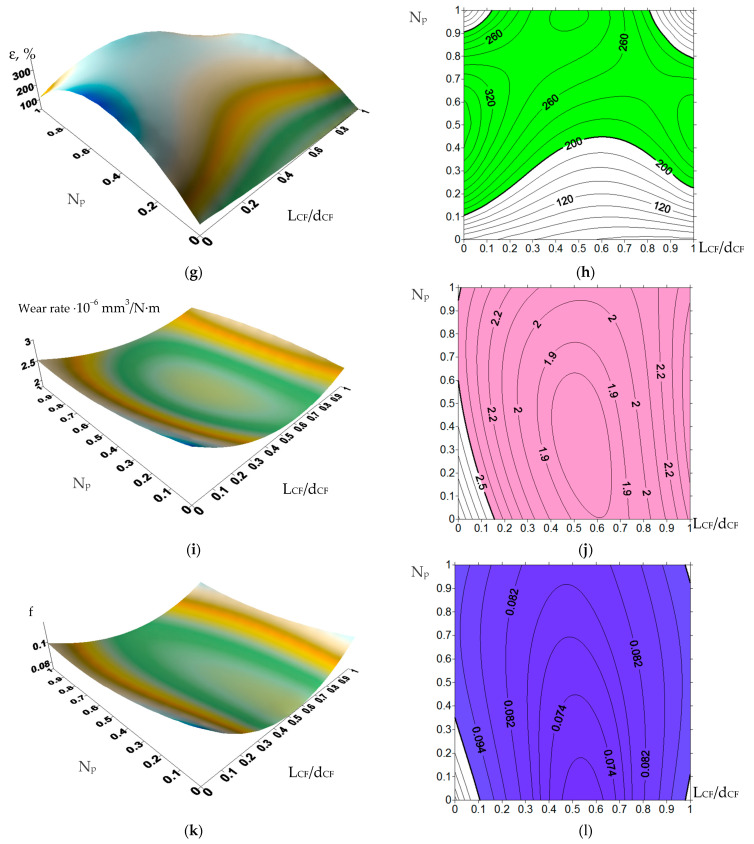

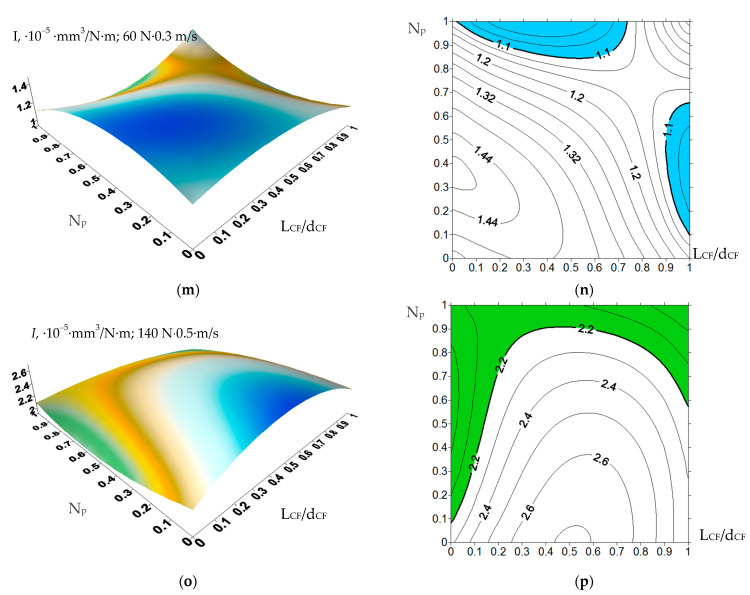
The functional properties of the UHMWPE-based composites vs. the number of processing passes in the extruder and the aspect ratio: (**a**,**b**) tensile Young’s modulus E (MPa); (**c**,**d**) ultimate tensile strength σ_T_ (MPa); (**e**,**f**) yield strength σ_Y_ (MPa); (**g**,**h**) elongation at break ε (%); (**i**,**j**) wear rate I (10^−6^·mm^3^/N·m) (the ‘ball-on-disk’ scheme; the ‘mild’ tribological conditions); (**k**,**l**) the friction coefficient f; (**m**,**n**) wear rate I·(10^−5^ mm^3^/N·m) (the ‘block-on-ring’ scheme; the ‘mild’ tribological conditions); (**o**,**p**) wear rate I·(10^−5^ mm^3^/N·m) (the ‘block-on-ring’ scheme; the ‘severe’ tribological conditions).

When drawing the isolines for all composite properties (Figure 12), the number of processing passes in the extruder and the aspect ratio of the glass filler were plotted along the X- and Y-axis, respectively. All node values were normalized from 0 to 1 to align the plots if different node points were used for various properties. The area that met the required limits was highlighted in color for each parameter.

**Figure 13:**
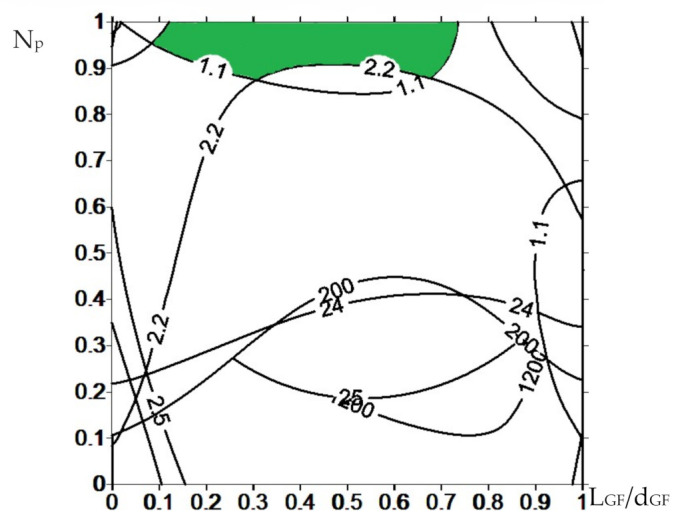
The area determining the number of processing passes and the aspect ratio of the glass filler required to obtain the UHMWPE-based composites with the predefined properties.

The overlaid areas of intersections of all graphs and isolines are shown in Figure 13. They determined the preferred number of processing passes in the extruder and the aspect ratio for the fabrication of the composites with the predefined properties. Recall that the processing passes in the extruder were not the number of sequential compounding, but the procedures associated with mixing in (i) the twin-screw extruder and (ii) FDM printing in the printer equipped with the micro-extruder.

According to Figure 13, the only composite loaded with MGF and fabricated by the FDM-TSE method was in the highlighted ‘green’ area.

This result was explained precisely by the possibility of uniform distribution of glass fibers in the polymer matrix, which enabled the achievement of high mechanical and tribological properties under the conditions of chemical adhesion (the presence of grafted VTMS groups). Loading UHMWPE with HGS (15–200 µm in size) did not allow being in the range of the specified parameters mainly due to the high wear rates (Figure 12m,n). On the other hand, CGF 3 mm in length protruded above the polymer matrix surfaces and had a scratching effect on the counterpart surface, which also caused the increase in wear rates. This excluded their use as the guides for friction units.

## 6. Conclusions

Based on the obtained results, the following conclusions were drawn.

It was experimentally determined that loading UHMWPE with 17% HDPE grafted with VTMS (trimethoxysilane) was able to bind 5% glass fillers of different aspect ratios, thereby determining the optimal mechanical and tribological properties of the composites. Further increasing the glass filler content resulted in the deterioration in their tribological characteristics due to insufficient adhesion of the extrudable matrix to the excessively loaded filler.

The multi-level approach was implemented to design the high-strength wear resistant ‘UHMWPE+17%HDPE-g-VTMS+12%PP’-based composites using the computer-aided algorithms. This resulted in the determination of the main parameters that provided their predefined mechanical and tribological properties and enabled us to assess the possible load-speed conditions for their operations in friction units.

The uniform distribution of the fillers in the matrix, the nature of the formed supermolecular structure and, as a consequence, the mechanical and tribological properties of the composites were achieved by optimizing the values of the main control parameters (the number of processing passes in the extruder and the aspect ratio of the glass filler).

For the fabrication of the composites, the twin-screw extrusion method was recommended for roller guides, plate chains, conveyors, and pipes for pumping corrosive liquids as well as 3D-printing (FDM) for complex-shaped parts operated in high-loaded friction units under various tribological conditions.

## Figures and Tables

**Table 1:** The fillers used for the fabrication of the composites.

Type	Mean Length (μm)	Diameter (μm)	The Aspect Ratio	Supplier
Hollow glass spheres (HGS)	15–200	15–200	1	3M, Saint Paul, MN, USA
Milled glass spheres (MGF)	200	9–14	20	Jushi Group Co. Ltd. Tonaxina Economic Development Zone, Zhejiang, China
Chopped glass spheres (LGF)	3000	9–14	300	Jushi Group Co. Ltd. Tonaxina Economic Development Zone, Zhejiang, China

**Table 2:** The mechanical properties of neat UHMWPE and the ‘UHMWPE+17%HDPE-g-VTMS+12%PP’-based composites loaded with hollow glass spheres (HGS).

Filler Composition (wt. %)	Density ρ (g/cm^3^)	Shore D Hardness	Young’s Modulus G (MPa)	Yield Strength σ_Y_ (MPa)	Tensile Strength σ_T_ (MPa)
None	0.934	57.7 ± 0.6	711 ± 40	21.6 ± 0.6	42.9 ± 3.1
Powder/Granules					
17%HDPE-g-VTMS+12%PP	0.933/ 0.939	57.8 ± 0.3/ 59.1 ± 0.2	876 ± 71/ 907 ± 75	25.1 ± 0.3/ 26.8 ± 0.6	19.1 ± 1.3/ 34.3 ± 2.7
+2.5% HGS	0.961/ 0.950	58.2 ± 0.7/ 58.5 ± 0.3	781 ± 23/ 1092 ± 28	23.2 ± 0.5/ 24.8 ± 0.3	19.1 ± 0.4/ 21.2 ± 1.6
+5.0% HGS	0.972/ 0.965	58.1 ± 0.5/ 58.2 ± 0.3	872 ± 72/ 1131 ± 39	23.2 ± 0.4/ 24.6 ± 0.1	18.4 ± 1.5/ 27.6 ± 0.5
+7.5% HGS	0.983/ 0.980	58.2 ± 0.4/ 58.8 ± 0.3	909 ± 52/ 1127 ± 54	23.1 ± 0.9/ 24.6 ± 0.4	21.6 ± 1.4/ 26.4 ± 1.6
+10.0% HGS	0.993/ 1.003	58.1 ± 0.5/ 58.7 ± 0.2	859 ± 82/ 1134 ± 85	22.1 ± 0.7/ 23.9 ± 2.2	18.2 ± 0.3/ 24.9 ± 3.9

**Table 3:** The mechanical properties of neat UHMWPE and the ‘UHMWPE+17% HDPE-g-VTMS+12% PP’-based composites loaded with MGF (200 µm in length).

Filler Composition (wt.%)	Density ρ (g/cm^3^)	Shore D Hardness	Young’s Modulus G (MPa)	Yield Strength σ_Y_ (MPa)	Tensile Strength σ_T_ (MPa)
None	0.934	57.7 ± 0.6	711 ± 40	21.6 ± 0.6	42.9 ± 3.1
Powder/Granules					
17%HDPE-g-VTMS+12%PP	0.933/ 0.939	57.8 ± 0.3/ 59.1 ± 0.2	876 ± 71/ 907 ± 75	25.1 ± 0.3/ 26.8 ± 0.6	19.1 ± 1.3/ 34.3 ± 2.7
+2.5% MGF	0.951/ 0.959	60.8 ± 0.5/ 58.6 ± 0.2	984 ± 82/ 1019 ± 38	24.8 ± 0.7/ 25.9 ± 0.8	20.2 ± 0.8/ 21.5 ± 2.2
+5.0% MGF	0.947/ 0.945	59.9 ± 0.6/ 60.4 ± 0.6	1063 ± 65/ 1153 ± 66	24.1 ± 1.9/ 26.5 ± 0.9	19.9 ± 0.5/ 25.3 ± 3.2
+7.5% MGF	0.978/ 0.972	60.8 ± 1.1/ 59.7 ± 0.2	1072 ± 55/ 1148 ± 87	25.3 ± 0.5/ 26.6 ± 0.5	20.6 ± 0.9/ 23.5 ± 1.6
+10.0% MGF	1.006/ 1.008	59.5 ± 0.7/ 59.4 ± 0.4	1101 ± 79/ 1155 ± 28	25.5 ± 0.6/ 26.7 ± 0.9	20.5 ± 1.3/ 22.3 ± 1.4

**Table 4:** The mechanical properties of neat UHMWPE and the ‘UHMWPE+17%HDPE-g-VTMS+12%PP’-based composites loaded with CGF (3 mm in length).

Filler Composition (wt.%)	Density ρ (g/cm^3^)	Shore D Hardness	Young’s Modulus G (MPa)	Yield Strength σ_Y_ (MPa)	Tensile Strength σ_T_ (MPa)
None	0.934	57.7 ± 0.6	711 ± 40	21.6 ± 0.6	42.9 ± 3.1
Powder/Granules					
17%HDPE-g-VTMS+12%PP	0.933/ 0.939	57.8 ± 0.3/ 59.1 ± 0.2	876 ± 71/ 907 ± 75	25.1 ± 0.3/ 26.8 ± 0.6	19.1 ± 1.3/ 34.3 ± 2.7
+2.5% CGF	0.979/ 0.964	57.9 ± 0.6/ 57.9 ± 0.3	1031 ± 50/ 1148 ± 67	20.9 ± 0.8/ 25.7 ± 0.5	18.3 ± 1.1/ 22.7 ± 1.7
+5.0% CGF	0.984/ 0.972	58.4 ± 0.4/ 58.9 ± 0.4	1208 ± 45/ 1241 ± 45	23.3 ± 0.9/ 25.2 ± 0.4	19.2 ± 1.5/ 25.2 ± 1.5
+7.5% CGF	1.009/ 0.999	58.4 ± 0.7/ 59.2 ± 0.4	1172 ± 21/ 1261 ± 50	24.1 ± 0.3/ 25.2 ± 0.3	18.9 ± 0.6/ 23.2 ± 3.1
+10.0% CGF	1.034/ 1.010	59.2 ± 0.5/ 59.4 ± 0.5	1233 ± 52/ 1276 ± 38	23.5 ± 1.1/ 24.9 ± 0.7	19.1 ± 0.7/ 22.5. ± 1.8

**Table 5:** The mechanical properties of the ‘UHMWPE+17%HDPE-g-VTMS+12%PP’-based composites.

Filler Composition (wt.%)	Density ρ (g/cm^3^)	Shore D Hardness	Young’s Modulus G (MPa)	Yield Strength σ_Y_ (MPa)	Tensile Strength σ_T_ (MPa)
0) 17%HDPE-g-VTMS +12%PP (HC-PM)	0.933	57.8 ± 0.3	876 ± 71	25.1 ± 0.3	19.1 ± 1.3
1) 17%HDPE-g-VTMS +12%PP (HC-TSE)	0.939	59.1 ± 0.2	907 ± 75	26.8 ± 0.6	34.3 ± 2.7
2) 17%HDPE-g-VTMS +12%PP (FDM-TSE)	0.925	58.4 ± 0.5	1210 ± 65	26.4 ± 0.6	22.7 ± 1.9
+5% HGS (HC-PM)/ +5% MGF (HC-PM)/ +5% CGF (HC-PM)	0.972/ 0.947/ 0.984	58.1 ± 0.5/ 59.9 ± 0.6/ 58.4 ± 0.4	872 ± 72/ 1063 ± 65/ 1208 ± 45	23.2 ± 0.4/ 24.1 ± 1.9/ 23.3 ± 0.9	18.4 ± 1.5/ 19.9 ± 0.5/ 19.2 ± 1.5
+5% HGS (HC-TSE)/ +5% MGF (HC-TSE)/ +5% CGF (HC-TSE)	0.965/ 0.945/ 0.972	58.2 ± 0.3/ 60.4 ± 0.6/ 58.9 ± 0.4	1131 ± 39/ 1153 ± 66/ 1241 ± 45	24.6 ± 0.1/ 26.5 ± 0.9/ 25.2 ± 0.4	27.6 ± 0.5/ 25.3 ± 3.2/ 25.2 ± 1.5
+5% HGS (FDM-TSE)/ +5% MGF (FDM-TSE)/ +5% CGF (FDM-TSE)	0.937/ 0.929/ 0.945	57.8 ± 0.5/ 61.5 ± 1.2/ 58.5 ± 0.7	1306 ± 42/ 1376 ± 71/ 1522 ± 58	26.9 ± 0.3/ 29.3 ± 0.3/ 27.9 ± 0.9	23.5 ± 0.4/ 29.9 ± 2.1/ 24.2 ± 0.3

**Table 6:** The tribological characteristics of neat UHMWPE and the ‘UHMWPE+17%HDPE-g-VTMS+12%PP’-based composites. The ‘pin-on-disk’ scheme.

Filler Content (%)	Wear Rate (10^−6^ mm^3^/N·m)			The Friction Coefficient ƒ		
None	2.72 ± 0.48			0.102 ± 0.003		
	powder	granule	3D-printing	powder	granule	3D-printing
17%HDPE-g-VTMS+12%PP	2.34 ± 0.14	2.64 ± 0.18	2.28 ± 0.23	0.093 ± 0.003	0.095 ± 0.003	0.096 ± 0.004
+5% HGS	3.02 ± 0.24	2.54 ± 0.32	2.52 ± 0.11	0.119 ± 0.009	0.098 ± 0.006	0.099 ± 0.003
+5% MGF	1.96 ± 0.06	1.84 ± 0.08	2.06 ± 0.15	0.073 ± 0.005	0.075 ± 0.004	0.079 ± 0.005
+5% CGF	2.32 ± 0.18	2.48 ± 0.22	2.37 ± 0.15	0.105 ± 0.006	0.097 ± 0.009	0.104 ± 0.003

**Table 7:** The tribological characteristics of neat UHMWPE and the ‘UHMWPE+17%HDPE-g-VTMS+12%PP’-based composites. The ‘block-on-ring’ scheme.

Filler Content (%)	Wear Rate (10^−5^ mm^3^/N·m) (Test Conditions—60 N·0.3 m/s)			Wear Rate (10^−5^ mm^3^/N·m) (Test Conditions—140 N·0.5 m/s)		
None	1.34 ± 0.21			3.30 ± 0.39		
	powder	granule	3D-printing	powder	granule	3D-printing
17%HDPE-g-VTMS+12%PP	1.27 ± 0.14	1.28 ± 0.05	1.25 ± 0.13	2.25 ± 0.09	2.10 ± 0.08	2.19 ± 0.20
+5% HGS	1.34 ± 0.15	1.45 ± 0.15	1.11 ± 0.07	2.28 ± 0.29	1.96 ± 0.17	2.09 ± 0.06
+5% MGF	1.40 ± 0.15	1.33 ± 0.06	0.96 ± 0.15	2.70 ± 0.17	2.49 ± 0.34	2.12 ± 0.14
+5% CGF	1.16 ± 0.10	1.04 ± 0.06	1.38 ± 0.08	2.31 ± 0.41	2.23 ± 0.05	1.91 ± 0.14

**Table 8:** The counterpart temperatures upon friction on neat UHMWPE and the ‘UHMWPE+17%HDPE-g-VTMS+12%PP’-based composites under various tribological conditions. The ‘block-on-ring’ scheme.

Filler Content, %	Temperature (°C) (Test Conditions—60 N·0.3 m/s)			Temperature (°C) (Test Conditions—140 N·0.5 m/s)		
None	31.4 ± 2			58.4 ± 2		
	powder	granules	3D-printing	powder	granules	3D-printing
17%HDPE-g-VTMS+12%PP	28.8 ± 2	29.4 ± 2	31.5 ± 2	49.5 ± 2	49.6 ± 2	52.5 ± 2
+5% HGS	26.4 ± 2	26.8 ± 2	31.0 ± 2	53.4 ± 2	50.5 ± 2	52.6 ± 2
+5% MGF	26.1 ± 2	26.6 ± 2	29.6 ± 2	52.1 ± 2	44.1 ± 2	43.2 ± 2
+5% CGF	28.8 ± 2	27.4 ± 2	31.7 ± 2	48.1 ± 2	50.8 ± 2	51.7 ± 2

**Table 9:** The properties of the materials fabricated by the ‘Misumi Group Inc.’ [42].

Properties	Values
Density (g/cm^3^)	0.94
Ultimate tensile strength (MPa)	44
Elongation at break (%)	450
Rockwell R Hardness	40
Izod impact strength (kJ/m)	>137
The friction coefficient under dry friction conditions	0.07–0.22
The friction coefficient under lubrication conditions	0.05–0.10

**Table 10:** The properties of the materials fabricated by ‘Guangzhou Engineering Plastics’ [43].

Properties	Values
Density (g/cm^3^)	0.94–0.96
Ultimate tensile strength (MPa) (23 °C)	22
Breaking strength (MPa)	42
Elongation at break (%)	600
Charpy impact strength (unnotched) (kJ/m^2^)	Without fracture
Shore D hardness	65
Abrasion resistance (Sand Slurry Test)	100
The friction coefficient	0.09–0.10

**Table 11:** The properties of the materials fabricated by ‘Polymer Industries’ [44].

Properties	Test Method	Values
Density (g/cm^3^)	[45]	0.932
Ultimate tensile strength (MPa)	[46]	24
Tensile Young’s modulus (MPa)	[46]	770 (112,000)
Elongation at break (%)	[46]	300
The friction coefficient (static)	[47]	0.2
The friction coefficient (dynamic)	[47]	0.15
Abrasion resistance	-	10
Izod impact strength (kJ/m)	[48]	>100
Shore D hardness	[49]	64

**Table 12:** The properties of the materials fabricated by ‘Tangyin Dingyuan Engineering Plastics Co. Ltd.’ [50].

Properties	Values
Density (g/cm^3^)	0.93–0.96
Average molecular weight (10^6^ g/mol)	3–10
Yield strength (MPa) (23 °C in air)	22
Ultimate tensile strength (MPa)	42
Charpy impact strength (notched) (mJ/mm^2^)	Without fracture
Ball indentation hardness (MPa)	42
Shore D hardness	65–70
Abrasion resistance	70–80 (100 on steel)
The friction coefficient (static)	≤0.16
The friction coefficient (dynamic)	≤0.10
Water absorption (%)	0
Elongation at break (%) (23 °C)	≥300

**Table 13:** The properties of the ‘Polystone^®^ M natural’ material fabricated by ‘Röchling Engineering Plastics’ [51].

Properties	Test Method	Values
Density (g/cm^3^)	[52]	0.93
Water absorption (%)	[53]	<0.01
Molecular weight (10^6^ g/mol)	-	~9
Yield strength (MPa)	[54]	20
Elongation at break (%)	[54]	>200
Tensile Young’s modulus (MPa)	[54]	680
Impact strength (kJ/m^2^)	[55]	Without fracture
Shore D hardness	[56]	63

**Table 14:** The properties of the ‘TIVAR^®^ 1000’ material fabricated by ‘Mitsubishi Chemical Advanced Materials’ [57].

Properties	Test Method	Values
Density (g/cm^3^)	[52]	0.93
Average molecular weight (10^6^ g/mol)	-	~5
Water absorption (%)	[53]	<0.1
Ultimate tensile strength (MPa)	[54]	19
Elongation at yield point (%)	[54]	15
Elongation at break (%)	[54]	>50
Tensile Young’s modulus (MPa)	[54]	750
Shore D hardness	[56]	60
Charpy impact strength (unnotched) (kJ/m^2^)	[55]	Without fracture
Charpy impact strength (notched) (kJ/m^2^)	[55]	115
The friction coefficient (dynamic)	[58]	0.15–0.30
Wear rate (μm/km)	[58]	8

**Table 15:** The properties of the ‘TIVAR^®^ 88’ material fabricated by ‘Mitsubishi Chemical Advanced Materials’.

Properties	Test Method	Values
Density (g/cm^3^)	[52]	0.93
Ultimate tensile strength (MPa)	[46]	40
Tensile Young’s modulus (MPa)	[46]	420 (61,000)
Elongation at break (%)	[46]	300
Shore D hardness	[49]	69
Izod impact strength (unnotched) (kJ/m)	[59]	Without fracture

**Table 16:** The properties of the ‘Werkstoff’S′^®^ PE 1000 ‘S′’ material fabricated by ‘Murtfeldt Kunststoffe GmbH & Co. KG’ [60].

Properties	Test Method	Values
Average molecular weight (10^6^ g/mol)	-	~5
Density (g/cm^3^)	[52]	0.93
Water absorption (%)	[53]	<0.01
Yield strength / Ultimate tensile strength, MPa	[54]	≥17/-
Elongation at break (%)	[54]	≥300
Tensile Young’s modulus (MPa)	[54]	700
Charpy impact strength (unnotched) (kJ/m^2^)	[55]	Without fracture
Charpy impact strength (notched) (kJ/m^2^)	[55]	≥170
Ball indentation hardness (MPa)	[61]	38
Shore D hardness	[56]	66
The friction coefficient under dry friction conditions	-	0.1–0.2

## Data Availability

The data presented in this study are available on request from the corresponding author.
